# Report of a Case of Incised Wound of the Upper Lid and Eye

**Published:** 1875-11

**Authors:** Richard M. Lewis

**Affiliations:** Savannah, Ga.


### Report of a Case of Incised Wound of the Upper Lid and Eye.

By RICHARD M. LEWIS, M.D., of Savannah, Ga.

On the morning of the 1st instant, Amelia Nicholls, colored, æt. twenty-four, having consulted my friend Dr. J. G. Thomas, on account of a severe injury of the left eye, was, as a case coming under my specialty, kindly sent to me by him for treatment.

Her history was that in a general melee across the river the night before, she had been suddenly struck in the left eye by a splinter of glass from a bottle, which an antagonist had shivered on the head of her husband, near whom she was standing.

Upon making an examination, I found that the sharp edge of the piece of glass, entering the upper lid about a quarter of an inch above and to the inner side of the outer canthus, had made a rather ragged cut entirely through it, and nearly parallel to its free margin, cutting off the upper edge of the tarsal cartilage to a point similarly situated as to the inner canthus, so that the lower fragment was only supported by a narrow band of tissue at each end. Gently lifting the lid, a truly pitiable sight was presented. A gaping wound in the eye extended from the outer margin of the cornea, through its horizontal diameter, the inner part of the iris, and almost entirely the ciliary muscle and bodies, to a point in the sclerotic, two or three lines from its inner edge, it being deepest first at the ciliary region, on account of the curvature of the globe.

The aqueous had all escaped, of course, but the lens was apparently in site, and there had been no loss of vitreous.

It is barely possible that by bringing the wound in the sclerotic together with one or two fine sutures, using atropine, cold applications, and a compress bandage, the eye could have been saved, but when I considered the great injury to the iris and ciliary region, particularly the unfavorable circumstances surrounding the patient, including her inaccessibility, which would prevent that close and unremitting attention, so essential to success, the seriously impaired vision, even with the best possible result, the great danger of sympathetic ophthalmia, and the fact that the first blush of inflammation had already made its appearance, and would almost certainly be followed by weeks of suffering, I unhesitatingly advised immediate removal of the eye.

The consent of the husband was with difficulty obtained, but he finally agreeing, I proceeded to operate, being kindly assisted by Drs. Duncan and Charlton, who fully concurred in my opinion.

The patient having been put under the influence of chloroform, the wóund in the lid was first closed with three sutures. A stop-speculum was then introduced, and the operation now almost universally performed by oculists for enucleation of the eye was done in the following manner:

Having made an incision through the conjunctiva, and subconjunctival tissue entirely around the cornea, and about a line from its margin, caught up the tendons of the recti muscles and divided them with the scissors close to the sclerotic. I next rotated the eye outward, and introducing behind it a pair of blunt pointed, curved scissors, cut the optic nerve, after which I turned the ball out of the socket, and divided the oblique muscles.

The operation being completed, a compress was applied to check the hemorrhage, and allowed to remain not quite an hour. Removing it at the end of that time, and finding that all bleeding had ceased, I sent the patient home, with direction to keep a cloth wet with cold water constantly applied over the closed lids. Next day she was doing well, and the same directions were continued, with the additional one of syringing out the socket gently two or three times daily, with tepid water to keep it clean. The day after that the wound in the lid was found to have entirely healed, but there being slight congestion and swelling of the conjunctiva, with a little discharge, principally of mucus. A weak solution of carbolic acid and sulphate of zinc was ordered in place of the tepid water. I saw her for the last time eight days after the operation, and the result was satisfactory in every respect. An excellent stump for an artificial eye, which moved with the good one, was made by the capsule of tedon, and the retracted mucles attached thereto.

Thus, by the prompt removal of an eye, which was practically already destroyed, there not being one chance in a hundred of preserving it, the patient was saved weeks of suffering, and then by no means improbable loss of the other organ.

That the sooner an eye lost by accident, and in certain cases by disease, is removed, the better for the patient, is an opinion generally accepted by ophthalmologists, and is certainly most heartily endorsed by me.

